# Anticancer selenopeptides from food sources: synthesis strategies and multitarget mechanisms

**DOI:** 10.1016/j.isci.2026.114895

**Published:** 2026-02-05

**Authors:** Mingyu Ma, Xiaotong Zhou, Xinyue Qiao, Linling Li, Shuiyuan Cheng, Yingtang Lu, Hua Cheng

**Affiliations:** 1 School of Modern Industry for Selenium Science and Engineering, Wuhan Polytechnic University, Wuhan 430048, China; 2 National R&D Center for Se-Rich Agricultural Products Processing, Wuhan Polytechnic University, Wuhan 430023, China

**Keywords:** biomolecular engineering, biotechnology, medical biotechnology

## Abstract

The dual challenges of drug resistance and toxicity in cancer therapy necessitate the development of new drugs with high efficacy and safety. Selenopeptides, which synergistically combine selenium’s redox regulation capabilities with the tumor-targeting specificity of peptides, represent a promising frontier in antitumor drug development. Based on the recent literature, this review summarizes the sources and preparation methods of selenium peptides, such as enzymatic hydrolysis and solid/liquid-phase synthesis. Furthermore, it elucidates their multitarget mechanisms of action, including the modulation of the PI3K/Akt signaling pathway, activation of immune cells, inhibition of angiogenesis, and induction of cancer cell apoptosis. Evidence from *in vitro*, *in vivo* and preliminary clinical studies confirms their effectiveness in inhibiting cancer cell proliferation and reducing tumor markers. This article reviews the current research progress to provide a comprehensive reference for the clinical translation and application of selenium peptides in cancer therapy.

## Graphical abstract

**Figure:**
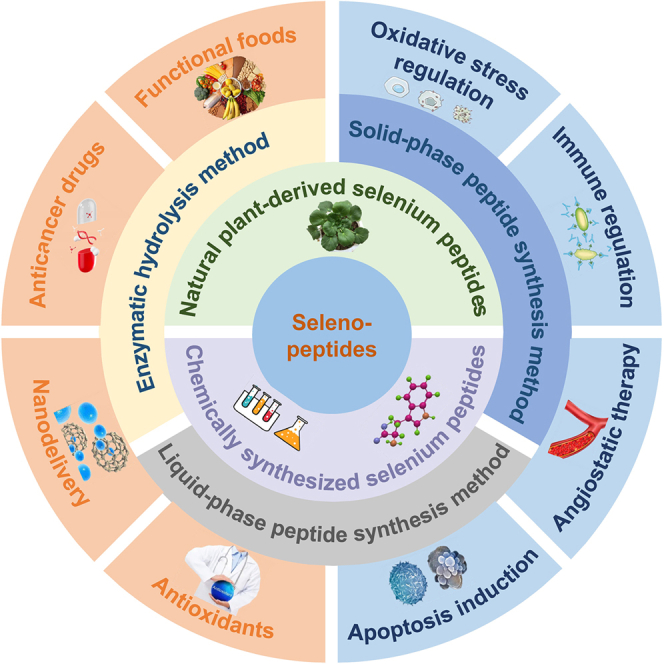

## Introduction

Cancer continues to pose a severe threat to human health,^1^ with its development and progression involving intricate physiological and pathological processes.^2^ Currently, the main anti-cancer therapies in clinical practice have significant shortcomings and are difficult to meet the treatment needs of high efficiency and low toxicity. For example, chemotherapeutic drugs: represented by cisplatin, can cause severe toxic and side effects such as nausea, vomiting, and leukopenia; targeted drugs: such as gefitinib, have a limited applicable population; immunotherapeutic drugs: represented by pembrolizumab, may have no response to treatment; selenium (Se) peptides, as new molecules that integrate the oxidative stress regulatory ability of Se and the tumor targeting of peptide chains, can specifically make up for the defects of existing therapies. Selenopeptides, a specific class of peptides containing Se,^3^ exhibit considerable potential for applications in cancer chemotherapy and biomedicine.^4^

Selenopeptides demonstrate multifaceted beneficial effects in cancer prevention and treatment.^5^ They can modulate the metabolic processes of cancer cells,^3^ influence energy metabolism pathways,^6^ and interfere with the rapid proliferation rate of cancer cells.^7^ Moreover, they possess significant antioxidant and free radical scavenging capabilities.^8^ During cancer development and progression, the proliferation of tumor cells generates excessive reactive oxygen species (ROS),^9^ which not only damage intracellular macromolecules but also promote cancer cell invasion and metastasis. Selenopeptides enhance the body’s intrinsic antioxidant defenses^10^ by increasing the activity of antioxidant enzymes. These enzymes include superoxide dismutase (SOD), catalase (CAT), and glutathione peroxidase (GPx).^11^^,^^12^ Additionally, selenopeptides can effectively scavenge excess ROS.^13^^,^^14^ This not only mitigates oxidative stress damage to normal cells but also inhibits the malignant transformation and progression of cancer cells.

Selenopeptides are a class of bioactive molecules that incorporate Se into short peptide chains,^15^ typically exhibiting relative molecular masses below several thousand Daltons. Structurally, they consist of short peptide chains composed of a limited number of amino acid residues, generally ranging from 2 to 20. This oligopeptide structure confers unique biological activities and metabolic properties. Se is embedded within the molecule, primarily in the forms of selenocysteine (Sec)^16^ or selenomethionine (SeMet).^17^ A common structural feature of selenopeptides is the substitution of sulfur atoms in specific amino acids with Se. This substitution generates selenoamino acids, which further participate in peptide chain formation.

Selenopeptides enhance the body’s immune surveillance and killing capabilities against cancer cells by modulating the immune system.^18^ They activate immune cells,^19^ such as T lymphocytes,^20^^,^^21^ B lymphocytes,^22^ and natural killer (NK) cells,^3^^,^^23^ promoting their proliferation and differentiation, thereby augmenting their ability to recognize and attack cancer cells. Furthermore, selenopeptides modulate the secretion of cytokines by immune cells, such as interleukins.^24^ By regulating cytokine secretion,^13^^,^^25^ they optimize the immune microenvironment,^14^^,^^26^ enhancing the inhibitory effect of the immune system on cancer cells.^3^

**Figure 1:**
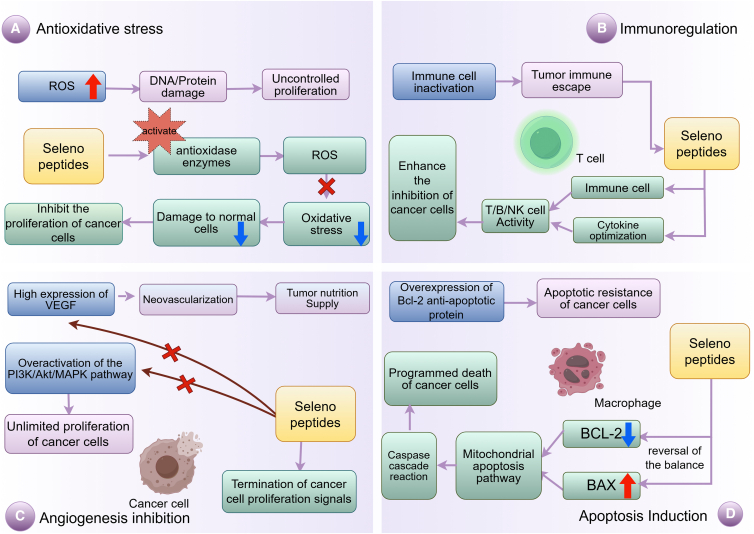
Mechanisms of anticancer action of selenopeptides (A) Antioxidant stress response mechanism. (B) Immunomodulatory mechanism. (C) Angiogenesis inhibition mechanism. (D) Apoptosis induction mechanism. ROS: reactive oxygen species; ACT: activation; BCL-2: B-cell lymphoma/leukemia-2 protein; BAX: BCL-2-associated X protein; The red “×” means blockage. The red upward arrow represents enhancement or promotion, while the blue downward arrow represents reduction or inhibition. The figure was created using Figdraw (https://www.figdraw.com).

Selenopeptides inhibit tumor angiogenesis through multiple mechanisms. Given that the proliferation, invasion, and metastasis of cancer cells rely on complex signaling cascades,^27^ these selenopeptides interfere with specific signaling pathways to block cancer cell growth and induce apoptosis. For instance, they inhibit the activity of key protein kinases within oncogenic signaling pathways, such as mitogen-activated protein kinases (MAPK),^28^ thereby suppressing pro-proliferative signal transduction in cancer cells.^11^ As shown in Figure 1 (mechanisms of anticancer action of selenopeptides), small molecule selenopeptides can exert anti-cancer effects through multiple pathways; this inhibitory effect is part of their multidimensional antitumor mechanisms.

Numerous studies have substantiated the anticancer activity of selenopeptides. *In vitro* cellular assays demonstrate their significant inhibitory effects on the growth of various cancer cell lines, including lung cancer,^3^ breast cancer,^29^^,^^30^^,^^31^ and colon cancer cells.^32^ *In vivo* animal studies using tumor-bearing models show that the administration of selenopeptides markedly reduces cancer growth rates, decreases cancer volume, and prolongs animal survival.^33^ These findings collectively underscore the considerable potential of selenopeptides in cancer prevention and therapy,^27^ positioning them as promising candidates for novel anticancer agents or adjuvant therapeutic strategies.

## Overview of selenopeptides

### Characteristics of selenopeptides

#### Fundamental concept and structural features

Selenopeptides are small-molecule oligopeptides or polypeptides with a molecular weight of less than 3 kDa, with a relatively simple structure.^34^ They can exist as degradation products of selenoproteins or be synthesized independently. For example, in plants, plants can absorb inorganic Se (selenate, selenite, and so forth) through their root systems,^35^ which is then catalytically converted into selenocysteine (SeCys) and selenomethionine (SeMet) by sulfidases and other enzymes.

These are then integrated into peptide chains through two pathways: The first is the SeMet pathway, in which SeMet is randomly incorporated into proteins through the methionine synthesis pathway, which is the main form of plant selenopeptides (such as soybean and rice selenopeptides). The second is the SeCys pathway: A few plants (such as Se-enriched *Cardamine violifolia*) can specifically insert SeCys through the UGA codon to form selenoproteins with active centers (such as glutathione peroxidase analogs).^36^

#### Pivotal role of selenium in anticancer activity

The incorporation of Se significantly enhances the bioactivity of selenopeptides,^37^^,^^38^ underpinning their substantial potential in the anticancer domain. Crucially, Se plays a central role in the anticancer mechanisms of these compounds.^39^ Aberrant metabolic processes in cancer cells generate excessive ROS,^40^ which damage intracellular macromolecules such as DNA, proteins, and lipids, thereby promoting tumor progression. Se is an essential core component of numerous antioxidant enzymes.^41^ Within selenopeptides, Se can serve as the active center or a cofactor for antioxidant enzymes, enhancing the activity of enzymes such as GPx.^42^ This accelerates ROS scavenging, mitigating oxidative stress-induced damage to cells and consequently controlling cancer cell growth and proliferation.

From a molecular biology perspective, Se modulates signaling pathways in cancer cells.^43^ For instance, it regulates the phosphatidylinositol 3-kinase/protein kinase B (PI3K/Akt) signaling pathway, which plays a critical role in cancer cell survival, proliferation, and invasion.^44^ In prostate cancer,^45^ Se can inhibit PI3K activity,^46^ impede Akt phosphorylation,^47^ leading to Akt inactivation, and induce apoptosis in prostate cancer cells. It can also induce apoptosis and autophagy in AGS gastric cancer cells by inhibiting the PI3K/Akt/mammalian target of rapamycin (mTOR) pathway.^48^ Furthermore, Se participates in regulating the expression and activity of cyclin-dependent kinases (CDKs) and cyclins, causing cancer cells to arrest at specific cell cycle phases and preventing uncontrolled proliferation.

### Sources and preparation of selenopeptides

**Figure 2:**
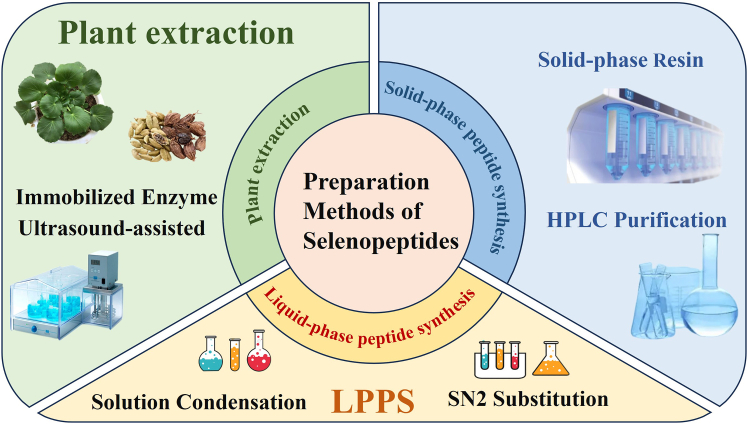
Three different types of sources and preparation methods of selenopeptides SPPS: solid-phase peptide synthesis; LPPS: liquid-phase peptide synthesis. This figure was created using Microsoft PowerPoint.

Selenopeptide can be obtained by extraction from natural Se-enriched plants^49^ or by chemical synthesis.^50^ As shown in Figure 2, there are three different types of Se peptide sources and preparation methods.

Due to the limitations of Se-enriched plant resources and Se content in the production of selenopeptides through natural extraction, it is difficult to achieve large-scale and standardized preparation of selenocysteine peptide segments. Thanks to its controllability and high efficiency, chemical synthesis technology has currently become the core method for obtaining a large number of selenopeptides with clear structures.^51^ Chemical synthesis allows precise control over the amino acid sequence and the specific site of Se incorporation within the peptide, enabling the targeted design of both structure and function. Through predefined amino acid sequences and Se linkage strategies, selenopeptides can be systematically synthesized.^52^^,^^53^ Chemically synthesized selenopeptides generally exhibit high purity and consistent quality, meeting the substantial demands of scientific research and clinical studies for these compounds. Common chemical synthesis methodologies include solid-phase peptide synthesis (SPPS)^54^ and liquid-phase peptide synthesis (LPPS).^52^

#### Natural extraction and modification from plant sources

For natural extraction, Se-enriched plants, including garlic^55^ and broccoli,^56^ represent major sources. Relative to non-accumulator species, these Se hyperaccumulators exhibit enhanced capacities for Se uptake and intracellular storage, predominantly in the form of organic Se compounds.^35^ This method utilizes Se-enriched plants (e.g., cardamom, *C*. *violifolia*) as starting materials. Proteins are first extracted from these plants, followed by hydrolysis using proteases. Zhu et al. demonstrated that immobilized alkaline protease hydrolysis of cardamom protein yielded Se-enriched peptides with high organic Se content.^57^

For extraction from Se-enriched plants, effective pre-treatment steps (e.g., washing, drying, and pulverization) are essential. Subsequently, suitable extraction solvents (e.g., water and ethanol) and methods such as ultrasound-assisted extraction, microwave-assisted extraction, and enzymatic extraction are employed to maximize yield and efficiency.^58^

#### Solid-phase peptide synthesis

SPPS involves the sequential elongation of the peptide chain on a solid support. Amino acids are coupled stepwise onto a suitable solid-phase resin according to the desired sequence^59^ (Table 1). Se is typically introduced at the designated position during the synthesis, either by incorporating selenoamino acid building blocks or through specific chemical modifications. Upon the completion of the chain assembly, the peptide is cleaved from the resin, and all protecting groups are removed. The crude product is then purified, often employing techniques such as high-performance liquid chromatography (HPLC), to yield the target selenopeptide with high purity.^53^ This method offers relative operational simplicity and is amenable to automation, making it suitable for larger-scale production. The foundational SPPS approach was first introduced by Merrifield.^60^ A key advantage is the facilitation of high yields, as unreacted reagents can be efficiently removed by washing after each coupling step, circumventing the need for chromatographic purification during intermediate stages.

**Table 1:** Comparison and optimization strategies for the chemical synthesis methods of selenopeptides

Dimension	Solid-phase peptide synthesis (SPPS)	Liquid-phase peptide synthesis (LPPS)	Optimization strategies	Literature
Synthesis process	Support activation → Amino acid coupling → Se embedding → Cleavage and purification	Solution condensation → Selenization reaction → Column chromatography separation	Microwave-assisted coupling, photocatalytic selenization	Xu et al.^51^; Dowman et al.^73^
Key reagents	Fmoc - amino acids, HATU, TFA, polystyrene resin	EDC/NHS, Na_2_Se_2_, β-chloropropionyl peptide	Immobilized enzyme technology, ionic liquid solvents	Ahmed et al.^52^; Dowman et al.^73^
Se embedding methods	Site-specific replacement of Cys with Sec on solid-phase carriers	Introduction of Sec through SN2 substitution reaction in solution	Site-selective photocatalytic modification	Zhu et al.^57^
Yield/purity	Yield 70%–90%, Purity >95%	Yield 50%–70%, purity 70%–85%	HPLC replaces column chromatography to improve purity	Kayrouz et al.^54^; Aravindhan et al.^65^
Scale/time consumption	From gram-scale to kilogram-scale, automated synthesis (24 h/10 - peptide)	Milligram to gram scale, manual operation (72 h/10 - peptide)	The automated liquid-phase synthesizer shortens the time	Aravindhan et al.^65^
Technical challenges	The carrier cost is high, and the operation is complex	Purification is cumbersome, and the yield of long peptides is low	Magnetic nanocarriers simplify separation	Dowman et al.^73^
Application scenarios	Pre - clinical drug production, long-peptide synthesis	Screening of new selenopeptides and synthesis of short peptides in the laboratory	Combined with the nano-delivery system	Li et al.^33^; Guan et al.^74^

This approach involves synthesizing the peptide first and then chemically introducing Se into the peptide chain. While operationally simpler, this method generally suffers from lower preparation efficiency, making it less suitable for large-scale industrial production.^57^ Through SPPS, selenopeptides containing specific tumor-targeting sequences (such as RGD and EGFR-binding peptides) can be customized. For example, the “Sec-Gly-Arg-Gly-Asp” targeting peptide can be synthesized and used to modify drug carriers such as liposomes and nanoparticles, thereby improving the delivery efficiency to tumor tissues (in preclinical studies, the tumor targeting of such modified carriers is more than 3 times higher than that of the unmodified group).^61^

**Figure 3:**
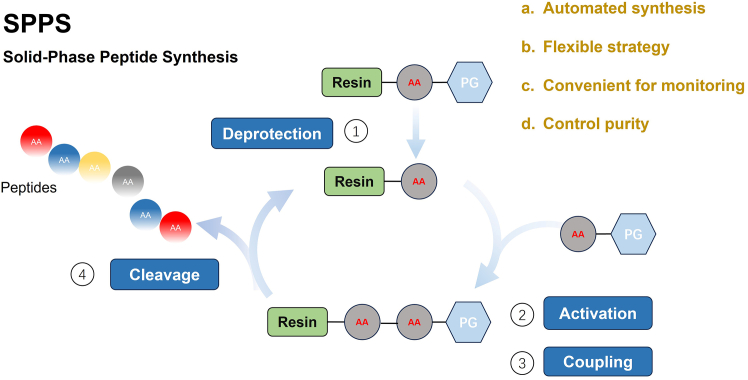
Solid-phase peptide synthesis The surface of the “Resin” contains active functional groups such as amino groups and hydroxyl groups, which covalently bind to the first amino acid through a linker, fixing it on the resin and providing an initial anchor point for peptide chain synthesis; “PG” is the abbreviation of Protecting Group, that is, a protecting group, which is a key group ensuring the precise synthesis of peptide chains; “AA” is the abbreviation of amino acid. The figure was created using BioRender (https://biorender.com).

Utilizing immobilized enzymes improves enzyme stability, reusability, simplifies downstream purification, and reduces costs.^62^ For instance, immobilizing alkaline protease on tannic acid and polyethyleneimine-modified nanoparticles for the preparation of *C. violifolia* selenopeptides demonstrated that the immobilized enzyme maintained high activity across a broad range of temperatures and pH values, exhibiting enhanced storage stability and reusability.^62^ As can be seen from Figure 3, it is the solid-phase peptide synthesis method.

#### Liquid-phase peptide synthesis

LPPS involves assembling the peptide chain in solution. The core principle for incorporating Se into the peptide relies on utilizing Sec or SeMet monomers or employing chemical reactions to introduce Se at specific sites within the peptide sequence synthesized in solution. This typically involves condensation reactions between functional groups of the Se-containing monomer and the activated groups of other amino acids or peptide fragments, enabling site-specific Se incorporation.^63^^,^^64^ LPPS offers the advantage of relatively milder reaction conditions but often requires more complex purification procedures post-synthesis. One approach was demonstrated by Aravindhan et al., who prepared Sec-containing peptides by reacting Na_2_Se_2_ with β-chloroalanine-based peptides.^65^ Various strategies for selenopeptide synthesis, particularly those involving Sec, have been reviewed by Muttenthaler and Alewood,^66^ Zhang et al.,^36^ Johansson et al.,^67^ and Pedrero and Madrid.^68^ Gieselman et al. reported novel synthetic routes for selenopeptides.^69^ Additionally, Tamura et al. described the synthesis of the Se-derived glutathione disulfide analog, glutamyl-Sec-gly (GSeSeG), via a solution-phase peptide synthesis process.^70^

**Figure 4:**
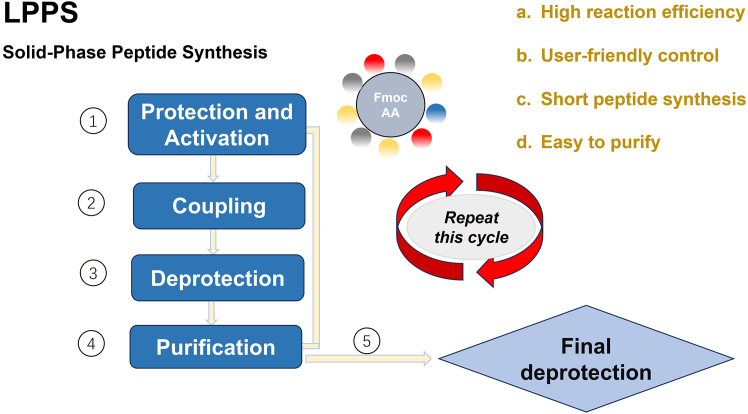
Liquid-phase peptide synthesis “Fmoc AA” is the abbreviation of Fmoc-amino acid, where “Fmoc” stands for 9-fluorenylmethoxycarbonyl, a commonly used amino-protecting group in peptide synthesis; “AA” is the abbreviation of amino acid. This figure was created using Microsoft PowerPoint.

Unique advantages of LPPS in synthesizing selenopeptides are that the solution-phase environment can reduce the non-specific binding of Se atoms to solid-phase carriers (a common problem in SPPS) and lower the isomerization rate of selenopeptides.^15^ As can be seen from Figure 4, it is the liquid-phase peptide synthesis method. Selecting appropriate techniques such as ultrafiltration^71^ and column chromatography^72^ is crucial to improve the purity of the isolated selenopeptides (Table 1).

## Anticancer activity of selenopeptides

### Mechanisms of anticancer activity

#### Modulation of cellular signaling pathways

Selenopeptides represent the primary form of Se in plants and animals and serve as crucial carriers of its physiological activity.^49^ Selenopeptides function as antioxidants, exhibiting strong nucleophilic characteristics and electron transfer capabilities.^26^^,^^73^^,^^74^^,^^75^ They reduce levels of ROS metabolites, directly or indirectly protecting cells from oxidative damage.^25^^,^^73^^,^^76^ Furthermore, they enhance the activity of antioxidant enzymes such as SOD and GPx, suppress the release of pro-inflammatory factors, and mitigate hepatic inflammatory responses, thereby demonstrating hepatoprotective effects.^77^

**Figure 5:**
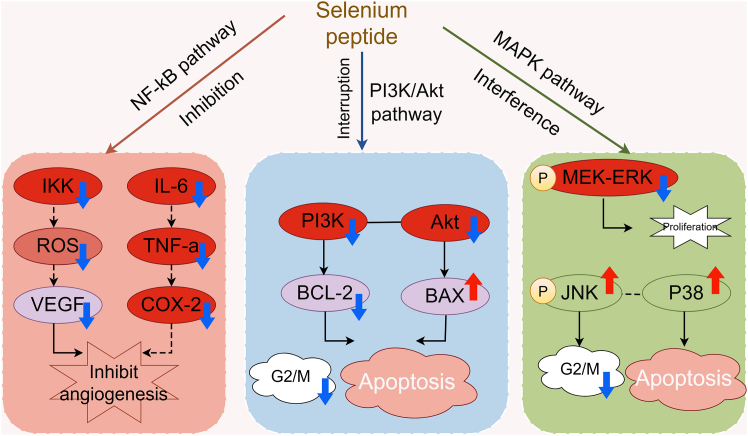
Modulation of major signaling pathways by selenopeptides P: phosphorylation; ROS: reactive oxygen species; BCL-2: B-cell lymphoma/leukemia-2 protein; BAX: BCL-2-associated X protein. The dashed line represents indirect regulation or synergistic effect, the solid arrow represents a direct regulatory relationship. The blue upward arrow represents enhancement or promotion, while the green downward arrow represents reduction or inhibition. The figure was created using Figdraw (https://www.figdraw.com).

The NF-κB pathway, a key regulator of inflammation and immunity, is frequently hyperactivated in cancer cells, resulting in the excessive secretion of pro-inflammatory factors. Selenopeptides reduce pro-inflammatory cytokine levels by inhibiting NF-κB pathway activation,^78^ thereby decreasing cancer cell invasion.^37^ Furthermore, they modulate multiple critical cellular signaling pathways to suppress tumor progression. Specifically, selenopeptides can inhibit both the PI3K/Akt and MAPK pathways.^79^ The MAPK pathway, exemplified by the ERK subfamily, transmits growth and stress-related signals. By interfering with the kinase phosphorylation cascade in this pathway, selenopeptides can block pro-proliferative signals in cancer cells and induce apoptosis (Figure 5).

Selenopeptides exert anti-cancer effects by regulating signaling pathways, activating immunity response, inhibiting angiogenesis, and inducing apoptosis.^80^ However, the realization of their *in vivo* efficacy depends on stable existence, reasonable metabolism, and efficient delivery. In terms of *in vivo* stability, oral selenopeptides are vulnerable to attack by gastrointestinal digestive enzymes (such as trypsin), with a degradation rate of 30%–50% within 2 h, and a plasma half-life of only 0.5–2 h.^13^ Small molecule selenopeptides are even more rapidly excreted due to glomerular filtration.^81^ In terms of metabolism, the liver is the core site, where selenomethionine methyltransferase and other enzymes catalyze their activation, transformation, or inactivation, and they are ultimately mostly excreted through the kidneys in the form of selenate, and so forth. To mitigate these challenges, nanocarriers can minimize enzymatic degradation through sustained release, exhibiting good biocompatibility yet high preparation costs; liposomes enable tumor targeting and toxicity reduction but suffer from low drug-loading capacity.^61^ These delivery systems offer promising strategies for enhancing the bioavailability of selenopeptides and facilitating their clinical translation.

#### Modulation of the cellular immune system

Selenopeptides exert anticancer effects by modulating the cellular immune system through three primary mechanisms.

**Immune cell activation.** Selenopeptides activate key immune cells, including T lymphocytes, B lymphocytes, and natural killer (NK) cells, stimulating their proliferation and differentiation.^22^ This enhances immune cells’ capacity to recognize and eliminate cancer cells.^19^ For instance, Z. Wei et al. demonstrated via co-culture experiments that selenopeptide-modified drug-loaded nanoparticles significantly increase NK cell cytotoxicity against cancer cells.^4^

**Cytokine secretion regulation.** Selenopeptides regulate cytokine secretion, including interleukins, to optimize the immune microenvironment. This involves suppressing the excessive production of pro-inflammatory factors (e.g., IL-6 and TNF-α) while enhancing the activity of anti-inflammatory cytokines. Consequently, tumor-associated inflammation is inhibited, indirectly suppressing cancer cell growth and metastasis.^26^

**Enhanced immune surveillance.** By improving the function of antigen-presenting cells (e.g., dendritic cells), selenopeptides promote efficient tumor antigen presentation, thereby activating adaptive immune responses. This strengthens the body’s specific recognition and clearance mechanisms targeting cancer cells.^18^

#### Inhibition of tumor angiogenesis

Tumor growth and metastasis depend on new blood vessels (angiogenesis) for nutrient and oxygen supply.^82^^,^^83^ Selenopeptides inhibit tumor angiogenesis through multiple mechanisms.^84^

**Suppression of VEGF.** They inhibit the expression and secretion of vascular endothelial growth factor (VEGF), a key promoter of angiogenesis. This is achieved by modulating relevant signaling pathways to reduce VEGF production in cancer cells.^85^

**Targeting endothelial cells (ECs).** Organic Se forms can significantly inhibit tumor angiogenesis by targeting thioredoxin reductase (TrxR), inducing apoptosis and cell-cycle arrest in ECs, and increasing ROS production within them. Inorganic Se forms also exhibit anti-angiogenic effects by inducing EC cycle arrest and elevating ROS levels. Collectively, this reduces the stimulatory effect on endothelial cells, thereby inhibiting their proliferation and migration and hindering new blood vessel formation.^86^

#### Induction of apoptosis

Inducing apoptosis in cancer cells is a crucial mechanism for the anticancer activity of selenopeptides.^87^ They activate intrinsic apoptotic signaling pathways, triggering programmed cell death in cancer cells.^88^

**Regulation of BCL-2 family proteins.** They modulate the expression of B-cell lymphoma-2 (BCL-2) family targets.^89^ This protein family includes anti-apoptotic members (e.g., BCL-2 and BCL-xL) and pro-apoptotic members (e.g., BAX and Bad). Selenopeptides disrupts the balance between these proteins, favoring apoptosis and playing a significant role in inhibiting cancer cell proliferation.^4^^,^^90^

**Cell cycle interference.** They interfere with the cell cycle progression of cancer cells, causing arrest at specific phases (e.g., G1/S or G2/M) and preventing normal cell division and proliferation.^91^ (Table 2).

**Table 2:** *In vitro* and *in vivo* anticancer activity of selected selenopeptides

Mechanism category	Specific action pathways	Key molecules/indicators	Experimental model/object	Data performance	literature
Antioxidant defense	Enhance the activities of GPx/SOD and scavenge ROS	GPx activity, ROS level	Mouse hepatocytes/Lung cancer A549 cells	GPx activity increased by 80%, and ROS decreased by 40%	Zhu et al.^12^
Immune activation	Activate T cells and enhance NK cell cytotoxicity	Infiltration of T cells, expression of perforin	Tumor-bearing mice/Peripheral blood mononuclear cells	The density of T cells increased by 60%, and the cytotoxicity increased by 65%	Hoffmann and Berry^19^
Angiogenesis inhibition	Downregulate VEGF expression and inhibit endothelial cell proliferation	VEGF protein, angiogenesis area	HUVEC cells/chick embryo chorioallantoic membrane model	VEGF decreased by 38%, and the vascular area decreased by 58%	Carmeliet and Jain^82^; Fu et al.^83^
Induce apoptosis of cancer cells	Regulate BCL-2 family proteins and activate the Caspase pathway	BAX/BCL-2 ratio, Caspase-3 activity	Breast cancer MCF-7 cells/Gastric cancer AGS cells	The ratio of BAX/BCL-2increased by 2 - fold, and the apoptosis rate increased by 55%	Zhu et al.^78^; Sanmartín et al.^86^
Inhibition of the NF-κB Pathway	Inhibit IKK kinase and reduce the secretion of pro-inflammatory factors	Nuclear translocation of IL-6, TNF-α, NF-κB	Colitis mice/breast cancer cells	IL-6 decreased by 45%, and the number of NF-κB nuclear positive cells decreased by 52%	Guo et al.^77^
Blockade of the PI3K/Akt pathway	Inhibit PI3K activity and activate PTEN phosphatase	*p*-Akt/Akt ratio, cell cycle distribution	Prostate cancer PC-3 cells	Akt phosphorylation decreased by 50%, G0/G1 phase arrest increased by 60%	Murdolo et al.^37^
MAPK pathway interference	Inhibit ERK phosphorylation and activate JNK/p38	ERK/JNK phosphorylation, apoptosis rate	Lung cancer A549 cells/tumor-bearing nude mice	ERK phosphorylation decreased by 42%, and the apoptosis rate increased by 55%	Wang et al.^28^; Zhu et al.^78^

### Validation of anticancer activity

Recent studies have substantiated the anticancer activity of selenopeptides through extensive *in vitro* and *in vivo* experimentation.

**Cellular studies.** Selenopeptides exhibit significant inhibitory effects on the proliferation of various cancer cell lines, including lung cancer,^3^ breast cancer,^29^^,^^30^^,^^31^ and colon cancer.^32^ Lin et al.^7^ demonstrated that selenopeptides isolated from *C. violifolia* reduce cancer cell viability in a dose- and time-dependent manner. They also suppress tumor cell proliferation and induce apoptosis.

**Animal models.** Animal experiments further validate the *in vivo* anticancer efficacy. Studies utilizing tumor-bearing mouse models, established by implanting human cancer cells, showed that treatment with selenopeptides led to significant antitumor effects. For instance, C. Li et al.^33^ reported that selenopeptides significantly enhanced the tumor-targeting efficiency of liposomes *in vivo*, with demonstrable differences observed within 24 h post-injection.

**Clinical evidence.** Although clinical trials involving selenopeptides remain relatively limited, emerging results indicate therapeutic promise. Demircan et al. found that selenopeptide treatment may elevate serum Se levels, subsequently influencing the expression or activity of selenoproteins and GPx.^92^ This mechanism enhances the body’s antioxidant capacity and modulates thyroid hormone metabolism, potentially contributing to observed reductions in tumor marker levels and tumor volume, highlighting its role in tumor suppression.

## Long-term toxicity of selenium

As an essential trace element for the human body, the normal functioning of Se’s physiological roles depends on an appropriate dosage. Long-term excessive intake can disrupt the metabolic balance in the body and trigger clear toxic reactions.^93^^,^^94^ The following is a systematic analysis from two aspects: the threshold of toxic dosage and the manifestations of long-term toxicity.

### The safe range of selenium intake

The toxicity of Se shows a significant “dose-dependent” characteristic. There is a clear limit to the human body’s tolerance range for Se, and exceeding this range may trigger toxicity. According to the recommendations of the World Health Organization (WHO) and the Chinese Nutrition Society, the recommended nutrient intake (RNI) of Se for adults is 50–200 μg per day, and the tolerable upper intake level is 400 μg per day.^95^ Within this range, Se can normally participate in the synthesis of selenoproteins such as GPx and TrxR, exerting antioxidant and immune regulatory effects without toxic risks. When the daily intake continuously exceeds 400 μg, the Se metabolic pathway in the body becomes saturated, and unmetabolized Se and its derivatives accumulate in tissues, gradually manifesting toxicity; if the daily intake exceeds 1000 μg, acute toxic symptoms may appear within 1–2 months, and long-term intake will lead to chronic organ damage.^93^

### Toxic manifestations of long-term excessive selenium

The earliest and most characteristic manifestations of Se excess include brittle hair with increased shedding, nail deformities (e.g., brittleness and white horizontal stripes), and cutaneous symptoms such as xerosis, pruritus, rash, or pigmentation changes. These symptoms serve as key clinical indicators for monitoring Se toxicity.^94^

Se excess primarily manifests as peripheral neuropathy, characterized by sensory disturbances (e.g., numbness, tingling, and hypoesthesia) in the extremities. In severe cases, it progresses to motor dysfunction, including muscle weakness and gait instability.^96^

Chronic Se overload irritates the gastrointestinal mucosa, resulting in gastrointestinal symptoms such as nausea, vomiting, abdominal pain, and diarrhea. Furthermore, as the primary organ for Se metabolism, the liver exhibits increased susceptibility to oxidative stress-induced hepatocellular damage under excessive Se exposure, leading to elevated serum transaminase levels-a hallmark of hepatocellular injury.^94^

## Factors influencing anticancer activity and optimization strategies

### Influencing factors

The anticancer activity of selenopeptides is dose-dependent.^97^ Studies suggest a recommended daily intake exceeding 200–250 μg to achieve cancer-preventive and inhibitory effects. The route of administration significantly impacts this activity,^5^ as it influences distribution, metabolism, and excretion. These processes further affect their bioavailability and activity at the target site. Furthermore, the anticancer potency varies across tumor types. Certain cancers, such as breast and lung cancer, exhibit greater sensitivity to selenopeptides, which demonstrate significant inhibitory effects on the growth and metastasis of these specific cancer cells.^68^

### Optimization strategies

Structural modification represents a key strategy for enhancing the anticancer activity of selenopeptides. Altering the mode of Se incorporation, peptide chain length, and amino acid composition can optimize their physicochemical properties and biological activity.^52^ Combining selenopeptides with other established anticancer agents is another effective approach to potentiate their efficacy, potentially through synergistic effects.^57^

## Application prospects and challenges of selenopeptide anticancer activity

### Application prospects

Selenopeptides demonstrate significant potential for applications in cancer prevention, diagnosis, therapy, and prognosis assessment, positioning them as promising candidates for novel anticancer agents. For high-risk populations (e.g., individuals with a family history of cancer or chronic exposure to carcinogenic environments), selenopeptides hold promise as effective preventive interventions.^11^ To realize this potential, a range of functional foods or nutraceuticals enriched with selenopeptides could be developed in the future. These products would be used for primary cancer prevention, to reduce malignant tumor incidence at its source.^80^ To further clarify the application value of selenopeptides, Table 3 compares their characteristics with those of existing clinical anticancer drugs.

**Table 3:** Comparison between selenopeptides and existing clinical anticancer drugs

Comparison index	Selenopeptides	Chemotherapy drugs (e.g., cisplatin)	Targeted drugs (e.g., gefitinib)	Immunotherapy drugs (e.g., pembrolizumab)	Literature
Mechanism of action	Multitarget (antioxidation, immune regulation, angiogenesis inhibition, and apoptosis induction)	Non-specific DNA damage	Single-target inhibition (e.g., EGFR)	Immune checkpoint blocking (e.g., PD-1)	Chen et al.^2^; Li et al.^11^; Chuai et al.^38^
Toxic side effects	Low (biocompatible and minimal damage to normal cells)	High (myelosuppression and gastrointestinal reactions)	Moderate (skin rash and diarrhea)	Moderate (immune-related pneumonia and colitis)	Chen et al.^2^; Kim et al.^98^
Drug resistance risk	Low (multitarget action)	High (multidrug resistance gene activation)	High (target mutation)	Moderate (tumor microenvironment remodeling)	Miao et al.^99^; Zakharia et al.^100^
Bioavailability	Low (prone to enzymatic degradation)	Moderate to high	High	High	Zakharia et al.^100^; Dobrzyńska et al.^101^
Applicable population	Broad (prevention in high-risk groups, adjuvant therapy	Narrow (limited to patients with tolerable toxicity)	Narrow (only for patients with specific mutations)	Narrow (limited response rate)	Chen et al.^2^; Wei et al.^4^; Dobrzyńska et al.^101^

As shown in Table 3, small molecule selenopeptides have obvious advantages in terms of low toxicity and low drug resistance risk, making them suitable for long-term prevention in high-risk populations and adjuvant therapy combined with other drugs. However, their low bioavailability is a key shortcoming compared with existing clinical drugs, which highlights the need for further optimization of drug delivery systems. However, challenges remain in addressing drug stability, bioavailability, and potential toxicity.

### Challenges and solutions

Drug stability poses a significant challenge in selenopeptide anticancer research. Selenopeptides are susceptible to enzymatic degradation and oxidation *in vivo*, leading to structural disruption and reduced activity.^101^^,^^102^ To address this, multiple strategies can be employed. On one hand, chemical modifications-such as introducing protective groups-can enhance resistance to enzymatic breakdown and oxidation. On the other hand, suitable drug delivery systems (e.g., liposomes and nanoparticles) can encapsulate selenopeptides, shielding them from the biological environment while enabling targeted delivery.

Low bioavailability is another major challenge for selenopeptides.^13^ Their gastrointestinal absorption may be compromised by various factors, resulting in insufficient effective doses entering systemic circulation. Additionally, toxicity is a critical factor in evaluating clinical viability.^103^ While current studies indicate good safety within therapeutic dose ranges, high doses may cause adverse effects^98^ and could potentially harm normal tissues and organs.^100^

## Conclusions and perspectives

This review systematically summarizes the research progress of small-molecule selenopeptides in antitumor therapy, with three core contributions: It clarifies the unique advantages of selenopeptides, combining Se’s antioxidant regulatory properties with the tumor-targeting capabilities of peptide chains, filling the gap in the development of low-toxicity, multitarget anticancer candidates. Second, it integrates the latest evidence from *in vitro*, *in vivo,* and preliminary clinical studies, confirming that selenopeptides inhibit the proliferation of cancer cells and reduce tumor marker levels.

Future research should focus on three key breakthrough areas: conducting large-scale clinical trials to verify the safety and efficacy of selenopeptides in different cancer types, and accelerating their translation from basic research to clinical application. Second, develop advanced delivery systems to improve bioavailability and *in vivo* stability. Third, elucidate the *in vivo* metabolic pathways and precise molecular targets of selenopeptides, laying a solid foundation for precision drug design.

## Acknowledgments

This research was supported by funding from the Science and Technology Major Program of Hubei Province, grant number (2025DJB079).

## Author contributions

Writing – original draft preparation and investigation, M.M.; conceptualization, methodology, and supervision, L.L. and Y.L.; writing –review and editing, X.Z.; validation and formal analysis, X.Q.; funding acquisition, H.C.; project administration, S.C. All authors have read and agreed to the published version of the article.

## Declaration of interests

The authors declare no competing interests.

## Declaration of generative AI and AI-assisted technologies in the writing process

During the preparation of this work, the author used DeepSeek to improve the readability and language of the article. After using the tool, the authors reviewed and edited the content as needed and take full responsibility for the content of the published article.
